# Journal of European Travels

**Published:** 1882-01

**Authors:** 


In "A Journal of Travels in Europe, during the Summer of 1881," Dr. C. Pearson gives a very readable little narrative, clearly showing that he was neither *halt*, *lame* nor *blind* while away!

